# Attitudes of Nursing Students in Saudi Arabia Toward Evidence-Based Research

**DOI:** 10.1155/nrp/1280246

**Published:** 2025-06-23

**Authors:** Hamad Dailah

**Affiliations:** Research and Scientific Studies Unit, College of Nursing, Jazan University, Jazan 45142, Saudi Arabia

**Keywords:** attitudes, evidence-based practice, medical education, nursing students, research, Saudi Arabia

## Abstract

**Background:** The integration of research into nursing practice is fundamental for advancing evidence-based healthcare. This study examines the factors influencing Saudi nursing students' attitudes toward evidence-based research.

**Methods:** A cross-sectional survey was conducted among nursing students in Saudi Arabia from September 2023 to January 2024. Data were collected via an online questionnaire, which included demographic details and the attitudes toward research (ATR) scale. Statistical analyses employed descriptive and inferential statistics.

**Results:** Of the 603 participants, 56.72% were male, predominantly aged 21–25 years (65.67%), and 76.12% attended public institutions. Most were pursuing a B.Sc. in nursing (85.07%). The overall mean score of the ATR scale was 3.13 ± 0.51 out of 5, indicating a moderately positive attitude level among the students. A significant negative correlation was found between research usefulness and research anxiety (*r* = −0.4, *p* < 0.001), and a strong positive correlation existed between research usefulness and positive ATR (*r* = 0.73, *p* < 0.001). Research anxiety was negatively correlated with positive ATR (*r* = −0.49, *p* < 0.001). Additionally, research difficulty was significantly correlated with both research anxiety (*r* = 0.78, *p* < 0.001) and positive ATR (*r* = −0.48, *p* < 0.001). Notably, a statistically significant association was observed between the type of institute and the overall mean score, with students from public institutes scoring higher than those from private institutes. Linear regression analysis further confirmed that the type of institute was a significant predictor of overall ATR.

**Conclusion:** The study revealed a moderately positive attitude among students toward the utility of evidence-based research in their profession while highlighting concerns in areas such as research anxiety and difficulty. Compared with their private institute counterparts, students from public institutes displayed more favorable attitudes, indicating a need for educational policymakers to address disparities in research education. Addressing research anxiety and perceived difficulty through curriculum design and teaching methods could foster more positive ATR among nursing students, contributing to the advancement of evidence-based practices in nursing care.

## 1. Introduction

Evidence-based practice (EBP) is a cornerstone of nursing, integrating the best available evidence with clinical expertise to improve patient care and outcomes [1]. For future nurses, particularly nursing students, acquiring the skills necessary for effective EBP implementation is essential. These skills include critical questioning, information retrieval, and literature critique [2, 3]. Nursing students must develop research competencies early in their education to locate, evaluate, and apply evidence-based research (EBR) effectively. However, many nursing students, like practicing nurses, find EBR difficult to access or disconnected from clinical practice, which reduces their confidence in applying EBP [4, 5]. To bridge this gap, nursing education must prioritize teaching EBR, focusing on increasing students' familiarity with research concepts throughout their studies [6]. The American Association of Colleges of Nursing (AACN) advocates for the inclusion of EBR in baccalaureate programs to equip nursing students with the skills necessary for evidence-based care [7, 8].

The importance of EBR in nursing education extends beyond academic theory, shaping the professional practice of nursing students and the future quality of patient care [2]. EBR allows students to adapt to evolving healthcare environments, diverse patient populations, and regulatory changes, all of which they will encounter in their professional careers [9]. Moreover, mastering EBR promotes the continuous advancement of the nursing profession, ensuring that students, as future healthcare providers, are capable of applying new research findings to improve clinical practice [1, 3].

Despite the recognized importance of EBR, nursing students' attitudes toward it remain a significant challenge. Research indicates that many undergraduate students hold negative views toward EBR, which impedes their engagement in research-related courses and undermines their willingness to participate in research during their education [10]. This negativity often stems from anticipatory anxiety, perceptions of research as tedious, and relief upon completing research courses [5]. Conversely, other studies suggest that some students recognize the value of EBR and maintain positive attitudes toward its role in professional nursing practice [10, 11]. Attitudes toward EBR tend to shift as students progress through their education, becoming either more or less favorable as their clinical experience increases [12]. Understanding these shifting attitudes is crucial for developing curricula that foster positive views toward research early in nursing education [13].

Given the critical role of EBR in nursing education, this study aims to investigate the attitudes of nursing students in Saudi Arabia toward EBR. By examining the key factors that shape their perspectives, this study offers insights for improving nursing education in the region. Understanding these attitudes is particularly important as there is limited research that includes nursing students across the country, from both public and private institutions, in the global literature on EBR. The findings provide valuable insights for improving educational policies and curricula in Saudi Arabia and contribute to global EBR literature. By cultivating a positive outlook on EBR and bolstering academic motivation, we can better prepare nursing students to become not only consumers of research but also contributors to it, ultimately advancing the profession as a whole.

## 2. Materials and Methods

### 2.1. Study Design and Population

This research employed a cross-sectional study design, conducted from September 2023 to January 2024 in Saudi Arabia. Our target population included both graduate and postgraduate nursing students. To determine the requisite sample size, we utilized G^∗^Power software [14]. Setting an effect size of 0.3, with an alpha level of 0.05 and aiming for a power of 0.80, the software indicated a minimum required sample size of 350 participants to achieve 80% power for detecting a medium effect size at the 0.05 significance level. We managed to collect data from a convenience sample of 603 respondents, which exceeded the minimum requirement and thus strengthened the study's validity. The data collection was conducted through an online questionnaire on Google Forms, with the survey link disseminated across various networks to effectively reach our intended audience.

### 2.2. Ethical Considerations

The study received approval from the institutional review board at Jazan University. Informed consent was obtained from all participants, ensuring that they were fully informed about the study's objectives, the confidentiality of their responses, and the voluntary basis of their involvement.

### 2.3. Data Collection Tool

The questionnaire comprised two main sections. The initial section collected demographic information, including gender, age, degree currently pursued, type of academic institute (public academic institutes are funded primarily by government sources, whereas private academic institutes rely on tuition and private funding), and prior research experience. The second section utilized the attitudes toward research (ATR) scale, a 30-item self-report measure developed and validated by Papanastasiou [15]. The ATR scale demonstrated a reliability coefficient of 0.94, indicating strong internal consistency among the scale items [16]. The participants rated each statement on a 1-to-5 scale, with one representing strong disagreement and five representing strong agreement. The ATR scale assesses five distinct factors: usefulness of research, research anxiety, research difficulty, life relevancy of research, and overall ATR. The “usefulness of research” factor, comprising nine items, delved into students' perceptions of the utility of research in their professional careers, assessing whether they viewed research as an essential tool in nursing practice. The “research anxiety” factor, with eight items, evaluated the levels of anxiety and stress students associated with research activities, a crucial aspect influencing their future engagement in research. The “research difficulty” factor, consisting of three items, aimed to uncover the challenges and obstacles students encountered in research, providing insights for necessary support and resources. Through four items, “life relevancy of research” was used to examine how students related research to their daily lives and experiences, assessing its perceived practical relevance. The “positive attitude toward research” factor, encompassing eight items, measured students' general feelings, enthusiasm, curiosity, and overall disposition toward research, capturing their intrinsic interest and engagement in research activities.

The response to each item ranged from 1 to 5, with higher scores indicating greater agreement. For the 13 negatively worded items, we reversed the scores for consistent interpretation across all items, and a higher number of responses on the Likert scale represented positive attitudes. We calculated factor scores by averaging responses to items within each factor and an overall mean score encompassing all five factors. The scoring system was categorized into three levels: “lower research attitude” (scores from 1.00 to 2.33), indicating lower engagement with research; “moderate research attitude” (scores from 2.34 to 3.66), reflecting an intermediate level of positive disposition toward research; and “high research attitude” (scores from 3.67 to 5.00), denoting a strong, positive attitude and awareness of the importance of research in nursing. This system helps in effectively assessing and classifying students' ATR.

### 2.4. Data Analysis

Data analysis was performed via IBM SPSS Statistics, Version 24.0. To explore differences in scores relative to participant characteristics, we applied one-way ANOVA and the independent samples *t* test. The Pearson correlation analysis was performed to examine the relationships among the five factors. A linear regression analysis was conducted to identify predictors of the overall ATR score, considering all the independent variables in a single model. A *p* value of less than 0.05 was set for statistical significance across all analyses.

## 3. Results

The demographic and educational characteristics of the 603 study participants are detailed in Table 1. The age group distribution revealed that 396 participants (65.67%) were aged between 21 and 25 years, 108 (17.91%) were aged 20 years and younger, and 99 (16.42%) were aged 26 years or older. The cohort included 261 females (43.28%) and 342 males (56.72%). A majority, 513 (85.07%), were undergraduate students enrolled in a B.Sc. in the nursing program, while 90 (14.93%) were engaged in postgraduate studies. The participants also varied in the type of institute they attended, with 459 (76.12%) from public institutes and 144 (23.88%) from private institutes.

The analysis of the overall ATR mean scores across demographic categories indicates some variations although not all are statistically significant. In terms of age, the 21–25 -year group had a slightly higher mean score (3.21 ± 0.54) than the other age groups did. Gender did not significantly influence the mean scores, with females scoring 3.10 ± 0.47 and males 3.16 ± 0.55. In terms of degree, postgraduates scored slightly lower (3.05 ± 0.48) than undergraduate students did (3.15 ± 0.53). Compared with those from private institutes (2.91 ± 0.43), students from public institutes presented a greater mean score (3.20 ± 0.53), and this difference was statistically significant (*p* value: 0.029). Participation in conferences, seminars, webinars, or workshops did not significantly affect the overall mean scores.

As shown in Table 2, an item analysis of the attitudes of nursing students toward EBR is performed across various dimensions, offering a perspective on their perceptions and sentiments. In the dimension focusing on research usefulness in the nursing profession, the highest level of agreement among students was observed for the item “Knowledge from research is as useful as writing,” with a notable 59.70% of students agreeing, culminating in a mean score of 3.72 ± 1.34. On the other hand, the item “Research should be indispensable in my professional training” garnered lower agreement, with only 35.82% of the students agreeing, reflecting in a mean score of 3.09 ± 1.32.

Regarding research anxiety, a moderate level of agreement was seen in the perception of research as complex, with 34.33% agreeing, as indicated by the mean score of 3.03 ± 1.23 for “Research is a complex subject.” However, the level of anxiety was evident, with the statement “Research scares me” receiving a prominent level of agreement at 32.84%, and the lowest mean score in this category was 2.75 ± 1.41.

In the positive ATR dimension, the highest score was for “The skills I have acquired in research will be helpful to me in the future,” with strong agreement from 55.22% of the students, leading to a mean score of 3.63 ± 1.31. In contrast, enjoyment of research had a lower score, with “I enjoy research” receiving a lower level of agreement (34.33%) and a mean score of 3.06 ± 1.24.

The relevance dimension showed that the students recognized the importance of research-oriented thinking in daily life, with 40.30% agreement that “Research-oriented thinking plays an important role in everyday life,” with a mean score of 3.19 ± 1.27. Nevertheless, some skepticism about the direct application of research in personal life was noted, with “Research is irrelevant to my life” resulting in substantial disagreement of 37.31% and a mean score of 2.96 ± 1.32.

In the research difficulty dimension, the highest mean score was for “I make many mistakes in research,” with 34.33% of the students acknowledging challenges in research practice, reflecting in a mean score of 3.12 ± 1.11. Understanding research concepts, however, was perceived as moderately difficult, with 35.83% agreement that “I find it difficult to understand the concepts of research,” with a mean score of 2.82 ± 1.13.

Table 3 presents the mean scores of different factors of the ATR scale. The highest mean score was observed for the factor of research usefulness (3.39 ± 1.19), with 44.78% of the students scoring in the high interval, indicating a strong perception of the importance of research. For positive ATR, the mean was 3.22 ± 1.07, with 32.84% in the high-level interval. Research anxiety had a mean of 2.89 ± 0.98, with 58.21% in the intermediate interval. The relevance in the life dimension score was 3.05 ± 0.62, with the majority (76.12%) in the intermediate interval. In terms of research difficulty, the mean score was 2.94 ± 0.98, with 50.75% in the intermediate interval. The research anxiety and research difficulty dimensions recorded the highest proportion of students, each with 23.88%, falling into the lower interval. Overall, the combined mean score for all factors was 3.13 ± 0.52, predominantly in the intermediate interval (80.60%), providing a comprehensive view of students' attitudes toward various research-related aspects.

In Table 4, the correlation matrix reveals significant correlations between the various dimensions of attitudes toward EBR among nursing students, providing quantitative insight into how different perceptions of research are interrelated among nursing students. A negative correlation was found between research usefulness and research anxiety (*r* = −0.4, *p* < 0.001), indicating that as the perceived usefulness of research increases, students' anxiety toward it decreases. A strong positive correlation was observed between research usefulness and positive ATR (*r* = 0.73, *p* < 0.001), suggesting that students who view research as useful also tend to have a more positive attitude toward it. Furthermore, there was a negative correlation between research anxiety and positive ATR (*r* = −0.49, *p* < 0.001), implying that greater anxiety is associated with less positive ATR. The correlation between research difficulty and research anxiety was also significant (*r* = 0.78, *p* < 0.001), suggesting that students who find research difficult experience higher anxiety levels. Additionally, a modest positive correlation was identified between relevance and research anxiety (*r* = 0.25, *p* < 0.05). Finally, a moderate negative correlation emerged between research difficulty and positive ATR (*r* = −0.48, *p* < 0.001), indicating that difficulty in research is correlated with less positive attitudes.

In Table 5, a linear regression analysis was conducted to identify predictors of overall ATR. The significant predictor included the type of institute, with students from private institutes scoring lower than those from public institutes (*β* = −0.64, *p*=0.019).

## 4. Discussion

The aim of the present study was to investigate attitudes toward EBR among students in nursing programs in Saudi Arabia, a critical aspect of nursing education that shapes future EBP. Research, as an integral component of nursing, necessitates a positive attitude and understanding from those who will soon be at the forefront of the healthcare industry [17–20].

The results of the ATR scale suggest that nursing students generally hold a moderate positive attitude toward EBR and view it as beneficial for their professional development. The highest mean score was observed for the factor of research usefulness (3.39 ± 1.19), indicating a strong perception among nursing students of the importance of research. This aligns with previous studies that highlighted the positive attitudes of nursing students toward EBR, emphasizing its role in promoting EBP [16, 18]. Research within the domain of nursing education has consistently indicated a positive attitude among students toward nursing research, underscoring the perceived usefulness of research in the nursing profession [21–23].

However, the dimensions of research anxiety and research difficulty recorded the highest proportion of students falling into the lower attitude interval, suggesting that these areas may pose challenges for students. The mean score for research anxiety was 2.89 ± 0.98, with a majority of students (58.21%) in the intermediate interval, suggesting that while students recognize the importance of EBR, they also experience a degree of anxiety related to it. This could be due to the perceived difficulty of research, as indicated by the mean score for research difficulty (2.94 ± 0.98). This finding is consistent with other research indicating that students often find research topics challenging to learn [10, 24]. Despite these challenges, the overall combined mean score for all factors was 3.13 ± 0.52, predominantly in the intermediate interval (80.60%), providing a comprehensive view of students' attitudes toward various research-related aspects.

The correlational analysis further elucidates these attitudes, revealing significant interrelationships among different perceptions of research. A negative correlation between research usefulness and research anxiety (*r* = −0.4, *p* < 0.001) suggests that increased perceptions of research utility are associated with reduced anxiety. A strong positive correlation exists between research usefulness and positive ATR factors (*r* = 0.73, *p* < 0.001), reinforcing the idea that valuing research correlates with a more favorable outlook. Conversely, higher anxiety and difficulty correlate with less positive attitudes (*r* = −0.49, *p* < 0.001, and *r* = −0.48, *p* < 0.001, respectively), and perceived research difficulty aligns with increased anxiety (*r* = 0.78, *p* < 0.001). These findings emphasize the need for continued efforts to foster positive attitudes toward EBR among nursing students while also addressing areas of concern such as research anxiety and perceived difficulty. In comparing the findings of this study with those of previous studies, a similarity lies in the recognition of the positive correlation between research usefulness and positive ATR as well as weak correlations between the usefulness of research and research anxiety (0.363) and between the usefulness of research and research difficulty (0.290) [15]. However, another study did not find a correlation between the usefulness of research and research anxiety or research difficulty [16]. These findings highlight the evolving dynamics of nursing education and the changing perceptions of students toward research, emphasizing the need for continual adaptation and a focus on reducing anxiety and perceived difficulty in research education.

This study reveals a significant difference in research attitudes between students from public and private institutions, with students from public institutions displaying more favorable attitudes (*p*=0.029). The linear regression analysis identified the type of institution as a significant predictor of research attitudes, with students from private institutions exhibiting lower attitude scores (*β* = −0.64, *p*=0.019). Previous research supports this difference, showing that public institution students tend to have more favorable perspectives [25, 26]. These differences may be attributed to variations in curricular focus, institutional support, or access to resources, highlighting the need for standardized research education across educational settings. Further investigation into the research environment and infrastructure at these institutions could provide valuable context, as universities with established research facilities and active research communities may foster more positive attitudes by reducing barriers to research engagement. Additionally, examining how students' career aspirations align with their research attitudes could offer deeper insights, as those pursuing careers in academia or research-intensive fields may have a stronger appreciation for research skills. Incorporating qualitative data from focus groups or interviews would further illuminate how educational experiences and institutional support influence attitudes, guiding the development of interventions to enhance research culture, particularly in private institutions where gaps in support and resources may exist.

In the item analysis of nursing students' attitudes toward EBR, the highest mean scores were observed in the dimension of research usefulness in the nursing profession, with the items “Knowledge from research is as useful as writing” (3.72 ± 1.34), “The skills I have acquired in research will be helpful to me in the future” (3.63 ± 1.31), “Research is very valuable” (3.42 ± 1.41), “Research is useful to every professional” (3.39 ± 1.39), and “I will employ research approaches in my profession” (3.39 ± 1.33), indicating a strong appreciation for the practical application and value of research among students. The benefits of involving students in research have been highlighted in previous studies [6, 27]. Moreover, the study revealed that the lowest mean scores were within the research anxiety and difficulty dimensions: “Research makes me nervous” (2.72 ± 1.35), “Research scares me” (2.75 ± 1.41), “Insecurity in research data analysis” (2.88 ± 1.25), “Research makes me anxious” (2.88 ± 1.26), “Trouble with arithmetic” (2.87 ± 1.22), “Research is stressful” (2.9 ± 1.27), “Difficulty understanding research concepts” (2.82 ± 1.13), and “Research process is complicated” (2.94 ± 1.23). This highlights a potential barrier in nurturing a research-oriented mindset among nursing students. Previous studies have revealed that students generally hold positive ATR while concurrently perceiving it as stressful [21]. To enhance nursing students' research orientation and address their anxiety and perceived difficulties, integrating practical research applications into the curriculum, emphasizing stress management and confidence-building in research abilities, strengthening instruction in research methodology, promoting mentorship programs, including real-world research projects, providing continuous feedback and support, and utilizing technology and innovative teaching tools are recommended. These strategies aim to align with students' recognition of the research's practical value, as indicated by high scores in the research usefulness dimension, while addressing challenges highlighted in the research anxiety and difficulty dimensions. Both enrolling in research courses and actively participating in research activities can positively affect students' awareness of and ATR [28, 29]. Furthermore, the necessity for the ongoing enhancement of quality services in nursing education has been emphasized.

### 4.1. Limitations

A limitation of this study is that it did not include certain demographic factors such as research experience, geographical location of institutions, and year of undergraduate study, which could have provided additional insights into their impact on students' ATR. Furthermore, academic study stress was not considered, which might also significantly influence these attitudes. The cross-sectional design of this study limits its ability to establish causality, only permitting the identification of associations at a single point in time. To address this limitation and verify the causal relationships among the observed factors, future research should consider a longitudinal study approach. Such an approach would enable the examination of these relationships over time, providing a more definitive understanding of causality and influence.

## 5. Conclusion

This study investigated the attitudes of nursing students in Saudi Arabia toward EBR, an essential element in nursing education influencing EBPs. The results indicated a moderate positive attitude, particularly in recognizing research's professional utility, with the highest mean score for research usefulness (3.39 ± 1.19). However, areas of concern included research anxiety and difficulty, where students showed lower attitudes, suggesting challenges in these domains. Despite understanding the importance of research, many students experienced anxiety, with a mean score of 2.89 ± 0.98 in this area. Correlational analysis revealed a negative association between research usefulness and anxiety and a positive link between research usefulness and overall positive ATR. In contrast, higher levels of anxiety and perceived difficulty were correlated with less positive attitudes. The study also revealed a significant difference in attitudes between students from public and private institutes, with public students showing more favorable attitudes. This is an important aspect for educational policymakers to consider in efforts to standardize research education across various types of institutes. The item analysis revealed high scores for recognizing the practical value of research but low scores for the dimensions of anxiety and difficulty, indicating a need for curriculum enhancements. To improve students' research orientation, the study suggests integrating practical research applications, emphasizing stress management, and enhancing instruction in research methodology. Participation in research courses and activities positively impacts students' attitudes, highlighting the importance of continual advancement in nursing education.

## Figures and Tables

**Table 1:** Demographic and educational characteristics.

Item	Variables	Count (%)	Overall ATR mean ± SD	*p* value
Age groups (years)	≤ 20	108 (17.91%)	2.84 ± 0.49	0.091
	21–25	396 (65.67%)	3.21 ± 0.54	
	≥ 26	99 (16.42%)	3.15 ± 0.33	

Gender	Female	261 (43.28%)	3.10 ± 0.47	0.662
	Male	342 (56.72%)	3.16 ± 0.55	

Degree currently pursuing	Postgraduate	90 (14.93%)	3.05 ± 0.48	0.648
	Undergraduate	513 (85.07%)	3.15 ± 0.53	

Type of institute	Public	459 (76.12%)	3.20 ± 0.53	0.029
	Private	144 (23.88%)	2.91 ± 0.43	

Conference, seminar, webinar, or workshop attendance	No	297 (49.25%)	3.23 ± 0.52	0.136
	Yes	306 (50.75%)	3.04 ± 0.50	

**Table 2:** Item analysis of attitudes toward research scale across various dimensions.

Items	Disagreement	Neutral	Agreement	Mean ± SD
I. Research usefulness in the nursing profession				
Research is useful for my career	171 (28.36%)	144 (23.88%)	288 (47.76%)	3.34 ± 1.41
Research is connected to my field of study	171 (28.36%)	135 (22.39%)	297 (49.25%)	3.37 ± 1.42
Research should be indispensable (essential) in my professional training	171 (28.36%)	216 (35.82%)	216 (35.82%)	3.09 ± 1.32
Research should be taught to all students	216 (35.82%)	126 (20.9%)	261 (43.29%)	3.13 ± 1.52
Research is useful to every professional	144 (23.88%)	153 (25.37%)	306 (50.75%)	3.39 ± 1.39
Research is very valuable	171 (28.36%)	144 (23.88%)	288 (47.76%)	3.42 ± 1.41
I will employ research approaches in my profession	153 (25.37%)	153 (25.37%)	297 (49.26%)	3.39 ± 1.33
The skills I have acquired in research will be helpful to me in the future	108 (17.91%)	162 (26.87%)	333 (55.22%)	3.63 ± 1.31
Knowledge from research is as useful as writing	108 (17.91%)	135 (22.39%)	360 (59.70%)	3.72 ± 1.34
II. Research anxiety				
Research makes me nervous	261 (43.28%)	189 (31.34%)	153 (25.38%)	2.72 ± 1.35
Research is stressful	207 (34.33%)	216 (35.82%)	180 (29.85%)	2.9 ± 1.27
Research makes me anxious	234 (38.81%)	171 (28.36%)	198 (32.84%)	2.88 ± 1.26
Research scares me	279 (46.27%)	126 (20.9%)	198 (32.84%)	2.75 ± 1.41
Research is a complex subject	171 (28.36%)	225 (37.31%)	207 (34.33%)	3.03 ± 1.23
Research process is complicated	216 (35.82%)	171 (28.36%)	216 (35.82%)	2.94 ± 1.23
Research is difficult	171 (28.36%)	243 (40.3%)	189 (31.35%)	3.03 ± 1.22
I feel insecure concerning the analysis of research data	216 (35.82%)	216 (35.82%)	171 (28.36%)	2.88 ± 1.25
III. Positive attitude toward research (positive research predisposition)				
I enjoy research	162 (26.87%)	234 (38.81%)	207 (34.33%)	3.06 ± 1.24
I like research	153 (25.38%)	207 (34.33%)	243 (40.30%)	3.22 ± 1.35
Research-acquired knowledge is as useful as arithmetic	144 (23.88%)	216 (35.82%)	243 (40.30%)	3.25 ± 1.25
Research is interesting	117 (19.40%)	216 (35.82%)	270 (44.78%)	3.42 ± 1.33
Most students benefit from research	153 (25.38%)	234 (38.81%)	216 (35.82%)	3.24 ± 1.26
I am inclined to study the details of research	135 (22.39%)	270 (44.78%)	198 (32.84%)	3.15 ± 1.21
IV. Relevance in life				
I use research in my daily life	216 (35.82%)	144 (23.88%)	243 (40.30%)	3.07 ± 1.28
Research-orientated thinking plays an important role in everyday life	189 (31.35%)	171 (28.36%)	243 (40.30%)	3.19 ± 1.27
Research thinking does not apply to my personal life	207 (34.33%)	207 (34.33%)	189 (31.34%)	2.99 ± 1.15
Research is irrelevant to my life	225 (37.31%)	171 (28.36%)	207 (34.33%)	2.96 ± 1.32
V. Research difficulty				
I have trouble with arithmetic	198 (32.83%)	234 (38.81%)	171 (28.36%)	2.87 ± 1.22
I find it difficult to understand the concepts of research	216 (35.83%)	243 (40.3%)	144 (23.89%)	2.82 ± 1.13
I make many mistakes in research	144 (23.88%)	252 (41.79%)	207 (34.33%)	3.12 ± 1.11

**Table 3:** Mean scores of different factors.

Variable	Mean ± SD	Lower interval count (%)	Intermediate interval count (%)	Higher interval count (%)
Research usefulness	3.39 ± 1.20	108 (17.91%)	225 (37.31%)	270 (44.78%)
Research anxiety	2.89 ± 0.99	144 (23.88%)	351 (58.21%)	108 (17.91%)
Positive attitude	3.22 ± 1.07	99 (16.42%)	306 (50.75%)	198 (32.84%)
Relevance in life	3.05 ± 0.62	54 (8.96%)	459 (76.12%)	90 (14.93%)
Research difficulty	2.94 ± 0.98	144 (23.88%)	306 (50.75%)	153 (25.37%)
Overall ATR	3.13 ± 0.52	36 (5.97%)	486 (80.60%)	81 (13.43%)

**Table 4:** Correlation matrix of research-related factors.

**Correlation matrix**	**Research usefulness**		**Research anxiety**		**Positive attitude**		**Relevance in life**		**Research difficulty**

Research usefulness	—								
Research anxiety	−0.4	⁣^∗∗∗^	—						
Positive attitude	0.73	⁣^∗∗∗^	−0.49	⁣^∗∗∗^	—				
Relevance in life	0.08		0.25	⁣^∗^	0.23		—		
Research difficulty	−0.34	⁣^∗∗^	0.78	⁣^∗∗∗^	−0.48	⁣^∗∗∗^	0.32	⁣^∗∗^	—

⁣^∗^*p* < 0.05.

⁣^∗∗^*p* < 0.01.

⁣^∗∗∗^*p* < 0.001.

**Table 5:** Linear regression analysis of predictors of attitudes toward evidence-based research.

Predictor	Estimate	SE	*t*	*p*
Intercept	3.01	0.26	11.63	< 0.001
Age groups (years)				
21–25–≤ 20	0.35	0.29	1.18	0.245
≥ 26–≤ 20	0.49	0.39	1.24	0.221
Gender				
Male–female	0.04	0.22	0.17	0.863
Degree currently pursuing				
Postgraduate–undergraduate	0.21	0.3	0.71	0.48
Type of institute				
Private–public	−0.64	0.27	−2.42	0.019
Conference, seminar, webinar, or workshop attendance				
Yes–no	−0.39	0.32	−1.22	0.228

## Data Availability

The data used to support the findings of this study are available on request from the corresponding author.
